# Role of magnetic resonance imaging in assessment of acetabular and femoral version in developmental dysplasia of the hip

**DOI:** 10.1590/0100-3984.2021.0133

**Published:** 2022

**Authors:** Karim Mohamed Elsharkawi, Mohamed Samy Barakat, Ali Abdel Karim Farahat, Amin Abdel-Razek Youssef Ahmed, Rim Aly Bastawi

**Affiliations:** 1 Alexandria University Faculty of Medicine, Alexandria, Egypt.

**Keywords:** Developmental dysplasia of the hip, Magnetic resonance imaging, Acetabulum/diagnostic imaging, Femur/diagnostic imaging, Bone anteversion/diagnostic imaging, Displasia do desenvolvimento do quadril, Ressonância magnética, Acetábulo/diagnóstico por imagem, Fêmur/diagnóstico por imagem, Anteversão óssea/diagnóstico por imagem

## Abstract

**Objective:**

To evaluate the role of magnetic resonance imaging (MRI) in the assessment of femoral and acetabular version in developmental dysplasia of the hip (DDH).

**Materials and Methods:**

This was a cross-sectional study of 20 consecutive patients with DDH (27 dysplastic hips) who were examined with MRI. In dysplastic and normal hips (DDH and comparison groups, respectively), we evaluated the following parameters: osseous acetabular anteversion (OAA); cartilaginous acetabular anteversion (CAA); femoral anteversion; osseous Mckibbin index (OMI); cartilaginous Mckibbin index (CMI); and the thickness of the anterior and posterior acetabular cartilage.

**Results:**

The OAA was significantly greater in the dysplastic hips. The CAA, femoral anteversion, OMI, and CMI did not differ significantly between the normal and dysplastic hips. In the DDH and comparison groups, the OAA was significantly lower than the CAA, the OMI was significantly lower than the CMI, and the posterior acetabular cartilage was significantly thicker than the anterior cartilage.

**Conclusion:**

Our findings confirm that MRI is a valuable tool for the assessment of femoral and acetabular version in DDH. Preoperative MRI evaluation has great potential to improve the planning of pelvic and femoral osteotomies.

## Abstract (pt)

**Objetivo:**

Avaliar o papel da ressonância magnética (RM) na avaliação da versão femoral e acetabular na displasia do desenvolvimento do quadril (DDQ).

**Materiais e Métodos:**

Estudo transversal de 20 pacientes consecutivos com DDQ (27 quadris displásicos) que foram examinados com RM. Nos quadris displásicos e normais (grupos DDQ e comparação, respectivamente), avaliamos os seguintes parâmetros: anteversão acetabular óssea (AAO), anteversão acetabular cartilaginosa (AAC), anteversão femoral, índice de Mckibbin ósseo (IMO), índice de Mckibbin cartilaginoso (IMC) e espessura da cartilagem acetabular anterior e posterior.

**Resultados:**

A AAO foi significativamente maior nos quadris displásicos. A AAC, anteversão femoral, IMO e IMC não diferiram significativamente entre os quadris normais e displásicos. Nos grupos DDQ e comparação, a AAO foi significativamente menor que a AAC, o IMO foi significativamente menor que o IMC, e a cartilagem acetabular posterior foi significativamente mais espessa que a anterior.

**Conclusão:**

Nossos achados confirmam que a RM é uma ferramenta valiosa para a avaliação da versão femoral e acetabular na DDQ. A avaliação pré-operatória por RM tem grande potencial para melhorar o planejamento das osteotomias pélvicas e femorais.

## INTRODUCTION

Acetabular version refers to the position of the acetabular cup in the axial plane^(1)^. It has been postulated that developmental dysplasia of the hip (DDH) is associated with excessive acetabular anteversion^(2-4)^. However, acetabular retroversion, due to reduced posterior coverage rather than increased anterior coverage, is seen in approximately 18% of cases of untreated DDH^(5-8)^. It is also a crucial measurement in the preoperative assessment of DDH cases scheduled for pelvic osteotomy, because the type of pelvic osteotomy will be determined by the site of acetabular deficiency (anterior or posterior). Most osteotomies, including Dega osteotomy, will correct insufficient anterior coverage^(9)^, whereas the correction of insufficient posterior coverage, caused by acetabular retroversion, requires certain other types of pelvic osteotomies. Correction of excessive anteversion helps restore the normal anatomy and biomechanics, as well as ensuring adequate femoral head coverage^(1,10)^. In contrast, undetected retroversion can lead to osteoarthritis, femoroacetabular impingement, and hip pain in adulthood^(11-14)^.

Acetabular anteversion, which is easily measured by computed tomography (CT), typically ranges from 15° to 20°^(1,15)^. However, CT measures only osseous acetabular anteversion (OAA). In pediatric patients, the acetabulum has cartilaginous portions, and cartilaginous acetabular anteversion (CAA) is therefore more representative of the true magnitude of acetabular anteversion. Magnetic resonance imaging (MRI) is superior to CT for the visualization of acetabular cartilage and thus for the determination of CAA. In addition, MRI does not expose patients to ionizing radiation, which has a major impact in pediatric patients^(1,16)^.

Femoral version is the angle between a line tangential to the chondral border of the posterior condylar axis and a line passing through the femoral neck axis. Femoral anteversion (FA) is an inward rotation of the axis of the femoral neck, relative to the femoral condyles, in the axial plane; it can be measured by MRI or CT. It ranges from 30° to 40° in children and decreases with age, typically being 13° in adults^(17)^. Abnormal FA is a risk factor for osteoarthritis^(15,18)^. There is controversy regarding the degree of FA in DDH, some studies having shown it to be increased^(19-21)^, whereas others have shown no such increase^(3,22)^. That can complicate the decision to perform femoral derotation osteotomy^(22)^.

The Mckibbin index (MI), also known as the Mckibbin instability index, is the sum of the angles of femoral and acetabular anteversion, that sum typically ranging from 30° to 60°. A normal MI is crucial for appropriate hip biomechanics^(23,24)^. There are few data in the literature regarding the MI in DDH; it may be normal, decreased, or increased^(25)^. High and low MIs are indicative of an increased risk of hip instability and femoroacetabular impingement, respectively^(26)^.

## MATERIALS AND METHODS

### Patient population

This was a cross-sectional study of 20 consecutive patients treated at the pediatric orthopedics and malformation clinic of our institution between July 2019 and December 2020. All patients were ≥ 2 years of age, had been diagnosed with DDH by X-ray, and were scheduled to undergo either triple pelvic osteotomy or combined femoral and Dega osteotomy. There were 27 dysplastic hips, collectively designated the DDH group, and 13 normal (contralateral) hips, collectively designated the comparison group. Patients who were candidates for closed reduction were excluded, as were those with cerebral palsy, those with traumatic hip dislocation, and those with hip dislocation due to sepsis.

### Examination method

All MRI examinations were performed in a superconducting, closed 1.5-T scanner (Signa Explorer; GE Healthcare, Milwaukee, WI, USA). Prior to each examination, patients were sedated by an anesthesiologist. Each examination was performed with a body coil and with the legs of the patient in the neutral position. We acquired an axial T1-weighted fast spin-echo (FSE) sequence, with a repetition time/echo time (TR/TE) of 423/10 ms, and an axial intermediate-weighted fat-suppressed proton density FSE sequence, with a TR/TE of 3,928/40 ms. For both FSE sequences, the following parameters were employed: field of view, 220 × 180 mm; matrix, 320 × 224 mm; echo train length, 16 ms; slice thickness, 3 mm; interslice gap, 0.3 mm; and number of excitations, 4. The scan time was 116 s for the T1-weighted sequence and 135 s for the intermediate-weighted sequence. We also acquired a three-dimensional (3D) spoiled gradient-echo (SPGR) sequence, with the following parameters: flip angle, 10°; TR/TE, 10/4 ms; field of view, 220 × 220; matrix, 224 × 224 mm; slice thickness, 1.6 mm; interslice gap, 0 mm; number of excitations, 2; and scan time, 164 s.

The total scan time was 453 s, with two localizers (18 s each), one for the T1-weighted sequence and the other for the rest of the sequences. For the intermediate-weighted fat-suppressed proton density FSE sequence and the 3D SPGR sequence, the scan range was from the iliac crests to the upper femurs. For the T1-weighted sequence, we used two image stacks with parallel imaging and the same parameters: the first was also from the iliac crests to the upper femurs, and the second was at the level of the knee joint, including the femoral condyles.

**Figure 1:**
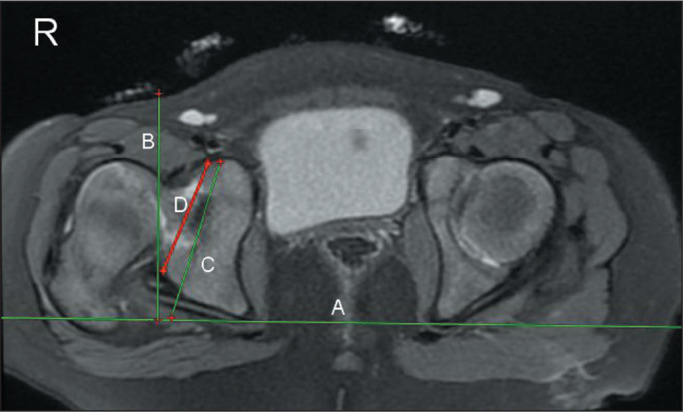
Axial intermediate-weighted fat-suppressed proton density FSE MRI sequence showing the lines employed for the measurement of OAA and CAA. Line A is tangential to the posterior aspect of the ischial tuberosities. Line B is orthogonal to line A. Line C is tangential to the outermost anterior and posterior bony rims of the acetabulum. Line D is tangential to the anterior and posterior chondrolabral junction. OAA is the angle between lines B and C. CAA is the angle between lines B and D. The right hip (R) is dysplastic, and the left hip is normal. Note that the femoral neck on the right side is at the same level as the femoral head on the left side, due to superior displacement of the dislocated femur on the right (dysplastic) side.

**Figure 2:**
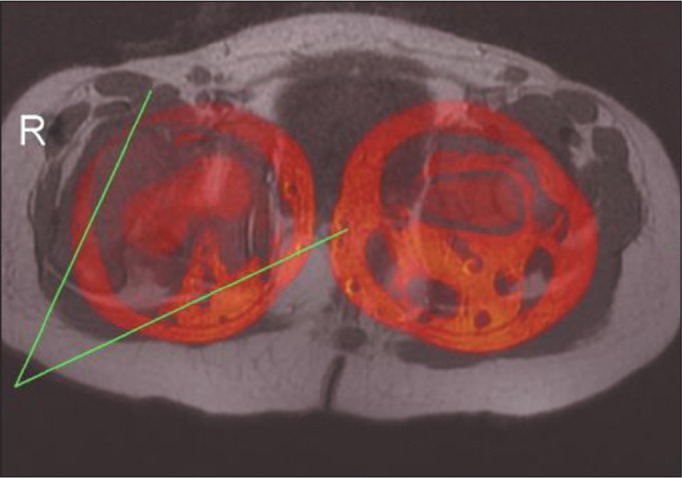
Fused axial T1-weighted FSE MRI sequence showing the lines employed for the measurement of FA in a dysplastic right hip (R). The femoral condyles on the right side are at the same level as the lower femoral diaphysis on the left side, due to superior displacement of the dislocated femur on the right (dysplastic) side.

**Figure 3:**
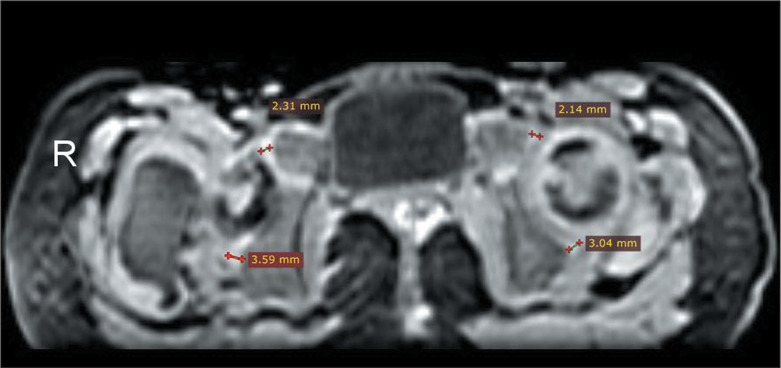
Axial 3D SPGR MRI sequence showing the cartilage thickness at the anterior and posterior rims of the acetabulum. The right hip (R) is dysplastic, and the left hip is normal. The femoral neck on the right side is at the same level as the femoral head on the left side, due to superior displacement of the dislocated femur on the right (dysplastic) side.

We measured OAA and CAA in a slice acquired at the midaxial point of the acetabulum (Figure 1). The FA value is determined by fusing two images, one acquired at the level of the femoral neck and the other acquired at the level of the femoral condyles (Figure 2). The osseous MI (OMI) is the sum of the OAA and FA values, whereas the cartilaginous MI (CMI) is the sum of the CAA and FA values. In an axial reconstruction of the 3D SPGR sequence (Figure 3), the cartilage thickness was measured at the anterior and posterior rims of the acetabulum. Measurements were performed by two musculoskeletal radiologists, with more than 5 and 10 years of experience, respectively, in musculoskeletal imaging.

### Statistical analysis

Data were analyzed with the IBM SPSS Statistics software package, version 20.0 (IBM Corp., Armonk, NY, USA). Qualitative data were described as absolute and relative frequencies. The Kolmogorov-Smirnov test was used in order to determine the normality of distribution. Fisher’s exact test was used in order to assess statistical significance, the level of which was set at 5%. The level of interobserver agreement was determined by using Pearson’s correlation test to calculate the intraclass coefficient (ICC) and was categorized as poor (ICC < 0.40), moderate (ICC 0.40-0.59), strong (ICC 0.60-0.79), or excellent (ICC ≥ 0.80).

## RESULTS

The mean age of the patients was 2.9 ± 1.12 years (range, 2-5 years). Of the 27 dysplastic hips evaluated, 24 (88.9%) were in female patients, three (11.1%) were in male patients, 21 (77.8%) were completely dislocated, and six (22.2%) were found to present only subluxation of the femoral head. All of the dysplastic hips were anteverted. The DDH was unilateral in 13 (65%) of the 20 patients and bilateral in seven (35%). Among the 13 patients with unilateral DDH, the right hip was affected in eight (61.5%) and the left hip was affected in five (38.5%). There were no statistically significant differences between the DDH and comparison groups regarding sex (*p* = 1.00) or mean age (*p* = 0.955).

Table 1 shows the OAA, CAA, FA, OMI, and CMI values, by group. The mean OAA value was significantly higher in the DDH group than in the comparison group (41.52 ± 5.55° vs. 23.15 ± 1.83°; *p* < 0.001). However, the CAA value did not differ significantly between the two groups (*p* = 0.326). The mean FA value was slightly higher in the DDH group than in the comparison group, although the difference was not statistically significant (*p* = 0.595). There were also no statistically significant differences between the two groups in terms of the OMI and CMI values (*p* = 0.418 for both). The OAA value was significantly lower than the CAA value in the DDH and comparison groups (*p* < 0.001 for both). In addition, the OMI was significantly higher than the CMI in both groups (*p* < 0.001 for both). The cartilage was significantly thicker at the posterior rim of the acetabulum than at its anterior rim, in both groups (*p* < 0.001 for both). There was no statistically significant difference between the DDH and comparison groups regarding the thickness of the cartilage at the anterior or posterior rim (*p* = 0.334 and *p* = 0.432, respectively). The overall interobserver agreement was strong (ICC = 0.7; 95% CI: 0.65-0.73).

**Table 1:** Values for OAA, CAA, FA, OMI, and CMI, in dysplastic hips (DDH group) and normal hips (comparison group).

Parameter	Group		P
	DDH (n = 27)	Comparison (n = 13)	
OAA (°), mean ± SD (range)	41.52 ± 5.55 (32.60-56.80)	23.15 ± 1.83 (18.50-24.90)	< 0.001
CAA (°), mean ± SD (range)	18.54 ± 4.56 (9.90-28.10)	16.85 ± 5.97 (7.70-26.20)	0.326
FA (°), mean ± SD (range)	38.49 ± 14.09 (12.30-78.60)	36.03 ± 12.48 (15.70-53.90)	0.595
OMI (°), mean ± SD (range)	54.70 ± 14.92 (25.60-97.0)	50.65 ± 14.05 (24.70-72.20)	0.418
CMI (°), mean ± SD (range)	57.0 ± 15.04 (27.90-98.20)	52.92 ± 14.18 (25.60-74.80)	0.418

## DISCUSSION

Many studies have assessed the degree of acetabular anteversion in cases of DDH. Li et al.^(27)^ used CT to assess OAA and found it to be significantly greater in dysplastic hips, as was the case in our study. Other authors have reported similar findings. In pediatric patients evaluated by MRI, Mootha et al.^(22)^ also found OAA to be significantly greater in dysplastic hips, as did Lu et al.^(1)^. In contrast, Duffy et al.^(21)^ found no statistically significant difference between dysplastic and normal hips regarding the OAA values determined from the MRI scans of pediatric patients. That difference could be explained by the fact that the mean age of the patients was lower in that study than in ours—7.6 months vs. 33.0 months (2.9 years). In patients with DDH, long-standing dislocation is associated with disease severity, which increases with age. A developmental defect of the anterior acetabulum, which is a recognized phenomenon after dislocation, results in a loss of the mutual stimulation between the femoral head and the acetabulum^(1)^.

Although we found CAA to be greater in dysplastic hips than in normal hips, the difference was not statistically significant, whereas Mootha et al.^(22)^ found it to be significantly greater in dysplastic hips. That difference could also be attributed to the aforementioned difference in age between their patient sample and ours. To our knowledge, there has been only one study assessing the progression of CAA in dysplastic hips over the long term. That study, conducted by Lu et al.^(1)^, showed that the CAA value was lowest in infancy and increased steadily up to the age of 2 years. Those authors also found CAA to be significantly greater in dysplastic hips than in normal hips. The difference in significance between our findings and those of Lu et al.^(1)^ could be explained by the fact that we employed a different study design, in which we used the contralateral (normal) hip for comparison. In addition, 22.2% of the hips in our DDH group were found to present only subluxation of the femoral head, rather than complete dislocation.

In the present study, the OAA value was found to be significantly lower than the CAA value, in dysplastic and normal hips. We attribute that to the fact that, in both groups, the cartilage was significantly thicker at the posterior rim of the acetabulum than at its anterior rim. However, there was no statistically significantly difference between the two groups in terms of the anterior or posterior cartilage thickness. These results corroborate those of other authors. Lu et al.^(1)^ also found that that the OAA value was significantly lower than the CAA value in dysplastic hips. They also categorized CAA as abnormal if it exceeded 21° after infancy, a value larger than the mean CAA value in our DDH and comparison groups.

Our findings differ from those of some other authors. Li et al.^(16)^ found no statistically significant difference between OAA and CAA in children with normal hips. That discrepancy could be explained by the difference between our two studies in terms of the ages of the patients, which ranged from 6 months to 16 years in the Li et al.^(16)^ study, compared with 2-5 years in our study. Albers et al.^(28)^ described changes in the posterior and anterior acetabular cartilage with the appearance of secondary ossification centers after the age of 9 years, which led to age-related changes in acetabular version. Lu et al.^(1)^ also found age-related variability in the speed of anterior and posterior endochondral ossification. In the present study, we used a 3D SPGR sequence, which has the advantage of providing a more accurate assessment of the cartilage thickness, thus informing decisions regarding orthotropic reconstruction.

We found no statistically significant difference between dysplastic and normal hips regarding FA. That is in keeping with the findings of other studies, such as that conducted by Sarban et al.^(3)^ who used CT to assess FA in children between 18 and 48 months of age. Mootha et al.^(22)^ also obtained similar results using MRI in children of similar age (12-48 months). In contrast, other studies—including those conducted by Akiyama et al.^(9)^ and Sugano et al.^(20)^—have shown significant excessive anteversion of the femoral neck in adults with a history of DDH. Many authors have suggested that FA increases with age in dysplastic hips, which could explain the variability across studies^(3,29)^. Such authors have stated that the primary pathology in DDH occurs at the acetabulum, and that femoral changes, such as excessive anteversion, are secondary adaptive phenomena. Therefore, excessive FA should not be encountered in very young individuals. These conclusions have a direct impact on management. In our opinion, derotation with the femoral osteotomy step of a triple pelvic osteotomy should not be routine and should be decided on case-by-case basis, as was concluded by Sankar et al.^(30)^.

The MI represents the sum of the angles of femoral and acetabular anteversion, the effects of which are thought to be additive^(15)^. Tönnis et al.^(15)^ studied 143 patients with various hip pathologies and found the MI to be low in all of those patients. In the present study, we found no statistically significant difference between dysplastic and normal hips in terms of the OMI. That discrepancy can be explained by differences in the patient selection process, because those authors included other hip pathologies, such as protrusio acetabuli, coxa vara, and coxa valga^(15)^. Similarly, we found no statistically significant difference between dysplastic and normal hips in terms of the CMI. That was an expected finding, given the lack of a significant difference between the two groups in terms of CAA and FA. Likewise, the OMI was statistically lower than the CMI in both groups, due to the similar relationship between the OAA and CAA values. To our knowledge, ours is the first study to assess the OMI and CMI in cases of DDH.

Our study has some limitations. First, we did not stratify the patients by age. That could represent a bias, given that femoral and acetabular anteversion may vary with age. In addition, our sample size was relatively small. Furthermore, because we deemed it unethical to recruit healthy subjects as controls, we used the contralateral (normal) hips for comparison purposes, which could also be interpreted as a limitation.

## CONCLUSION

The results of this study underscore the importance of MRI in the preoperative assessment of femoral and acetabular version in cases of DDH. The advantage of preoperative MRI for surgical management is that it can preclude the need for routine femoral derotation. In addition, MRI has the advantage of allowing the visualization of the cartilaginous framework of the hip. The visualization of the acetabular cartilage by MRI is important in that the apparent increase in OAA is not clinically relevant and should not be taken into account, because the true CAA is not significantly increased in dysplastic hips.
